# Human TNF-Luc reporter mouse: A new model to quantify inflammatory responses

**DOI:** 10.1038/s41598-018-36969-x

**Published:** 2019-01-17

**Authors:** Faisal Minshawi, Mike R. H. White, Werner Muller, Neil Humphreys, Dean Jackson, Barry J. Campbell, Antony Adamson, Stamatia Papoutsopoulou

**Affiliations:** 1 Faculty of Biology, Medicine and Health, School of Biological Sciences, Manchester, M13 9PT United Kingdom; 2 Department of Laboratory Medicine, Faculty of Applied Medical Science, Umm Al-Qura University, Makkah, Saudi Arabia; 3 Department of Cellular and Molecular Physiology, Institute of Translational Medicine, University of Liverpool, Liverpool, L69 3GE United Kingdom

## Abstract

Tumour necrosis factor (TNF) is a key cytokine during inflammatory responses and its dysregulation is detrimental in many inflammatory diseases, such as rheumatoid arthritis and inflammatory bowel disease. Here, we used a bacterial artificial chromosome (BAC) construct that expresses luciferase under the control of the human *TNF* locus to generate a novel transgenic mouse, the hTNF.LucBAC strain. *In vitro* stimulation of hTNF.LucBAC cells of different origin revealed a cell specific response to stimuli demonstrating the integrated construct’s ability as a proxy for inflammatory gene response. Lipopolysaccharide was the most potent luciferase inducer in macrophages, while TNF was a strong activator in intestinal organoids. Lipopolysaccharide-induced luciferase activity in macrophages was downregulated by inhibitors of NF-κB pathway, as well as by Interleukin-10, a known anti-inflammatory cytokine. Moreover, the transgene-dependent luciferase activity showed a positive correlation to the endogenous murine soluble TNF secreted to the culture medium. In conclusion, the hTNF.LucBAC strain is a valuable tool for studying and screening molecules that target TNF synthesis and will allow further functional studies of the regulatory elements of the *TNF* locus.

## Introduction

Tumour necrosis factor (TNF) is an important pro-inflammatory cytokine produced by the majority of the cells of the immune system. It plays a crucial role in homeostasis and disease pathogenesis^1^. Uncontrolled release of biologically active TNF is linked to development of inflammatory and autoimmune diseases like rheumatoid arthritis, inflammatory bowel disease (IBD), psoriasis and ankylosing spondylitis^1^. At the DNA level, the coding region of human *TNF* gene is located within *TNF/LT* locus, together with the Lymphotoxin-α (*LTA*), and Lymphotoxin-β (*LTB*) genes^2^. The *TNF* gene is one of the immediate early genes and there is a wide range of stimuli that activate its transcription. Such examples are the calcium-dependent pathway^3^, bacteria (such as *Mycobacterium tuberculosis*) and viruses^4,5^, osmotic stress^6^ and TNF itself ^7^. The transcription of the *TNF* gene is regulated by the enhanceosome, a higher-order protein structure, which in the case of the *TNF* promoter, assembles in a cell-type and stimulus-specific manner^8^. For example, calcineurin phosphatase mediates the induction of human TNF in stimulated T- and B-lymphocytes^3^. However, in macrophages, lipopolysaccharide (LPS) induces human TNF production by activation of extracellular signal-related kinase (ERK) activity^9^. The NFAT, ATF-2/Jun, Ets/Elk, and Sp1 protein along with CEB/p300 play a crucial role in regulation of TNF expression^10^. Moreover, there is a number of constitutive and inducible DNase I hypersensitive sites (HSs), which have been mapped across the *TNF/LT* locus. Barthel and colleagues showed that intron 3 in the human *TNF* gene contains a constitutive HSs site in Jurkat T cells, but not in THP-1 monocytic cells, demonstrating cell-type specificity of different regulatory regions^11^. Therefore, the human *TNF* gene has multiple transcription-binding sites that act in a cell-type and stimulus-specific fashion. In 1991, the first human TNF transgenic mouse was reported and revealed a major impact of TNF in chronic inflammatory polyarthritis^12^. Since then, many studies have utilised TNF transgenic mice in order to evaluate the pathways in rheumatoid arthritis^13^ and many studies focused on anti-TNF therapies^14^. Due to complex genomic organisation and regulation of the *TNF* gene^15^, it is vital that any reporter system should include all potential regulatory elements, and in the correct context to faithfully recapitulate and accurately report on *TNF* gene activation in different cell-types. Reporter constructs are created by placing a suitable genetically encoded reporter gene under the control of the regulatory elements of the gene of interest. Classically, plasmids and viruses have been widely used, but are limited by the size of genetic material that can be included. However, bacterial artificial chromosomes (BAC) encompass large (~150–200 kb) genetic regions, that can be propagated and manipulated *in vitro* by recombineering strategies to integrate a reporter gene^16^. The *TNF* genetic region is complex with respect to gene activation, thus the use of a BAC would ensure the inclusion of all such elements. Furthermore, the use of a human BAC on a mouse background allows for the study of human gene activation in a surrogate physiological system. Genetically encoded reporters, such as luciferases and fluorescent proteins, are powerful tools to detect gene expression, providing both accurate and quantifiable dynamic measurement of activity in gene expression. Firefly (*Photinus pyralis*) luciferase is particularly suitable for use *in vivo* due to its short half-life (40 min for destabilised Luc), high signal to noise ratio, high dynamic range and favourable pharmacokinetics of its luciferin substrate. The emission spectrum is shifted slightly towards red, often enabling direct *in vivo* assay^17^. Moreover, it is sensitive reporter with a wide dynamic range, a short-assay time and is non-radioactive. In this study, we have generated a reporter mouse by utilising a BAC that carries the firefly luciferase coding sequence under the control of the human *TNF* promoter. This system allowed us to directly measure human *TNF* promoter activity in *ex-vivo* and *in vitro* approaches.

## Results

### hTNF.LucBAC reporter construct

**Figure 1:**
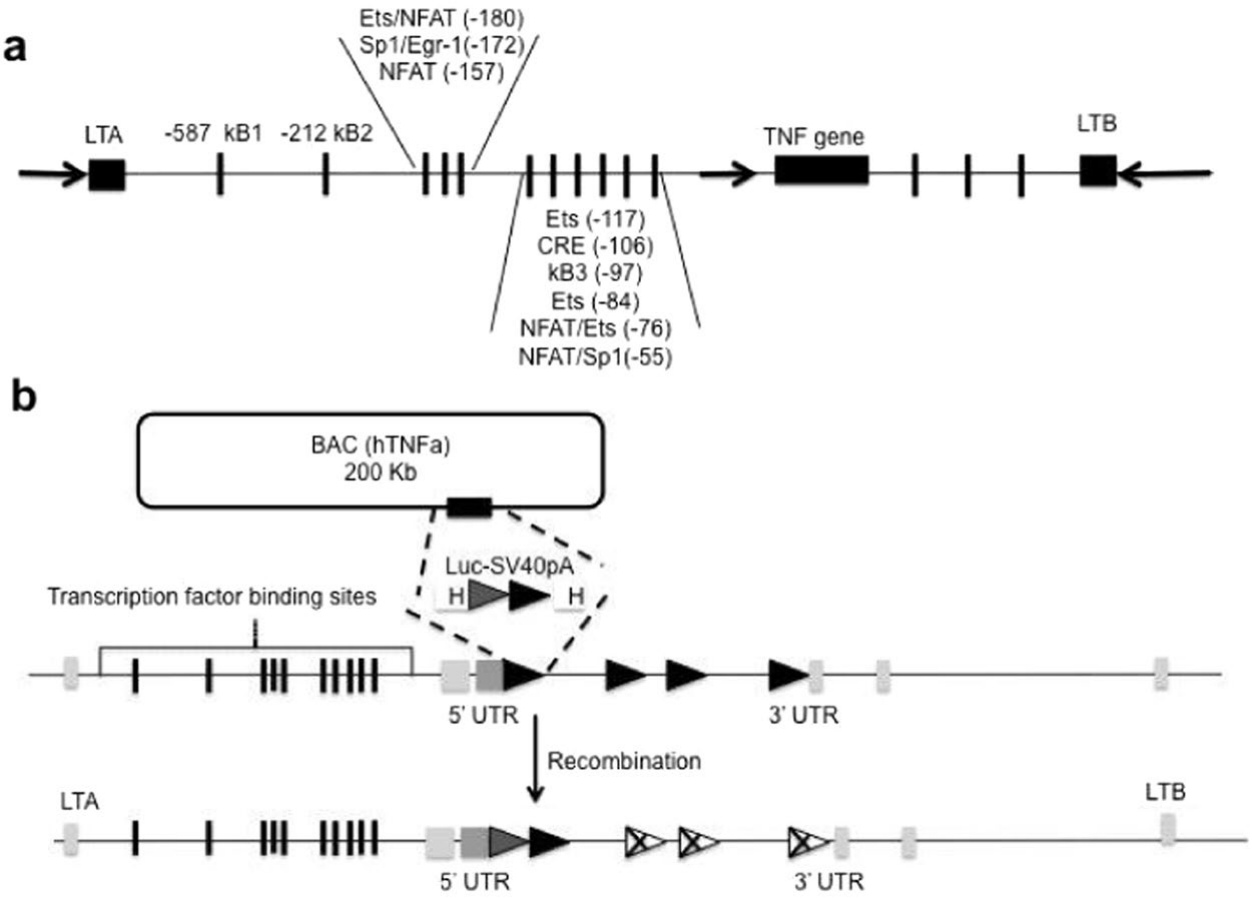
Schematic illustration of the human *TNF* locus and generation of the hTNF.LucBAC construct. Transcription factor binding sites within the human *TNF* promoter region (**a**). A BAC construct containing all the regulatory elements within the human *TNF* locus was used as a template for genetic recombination of the *TNF* gene. The firefly luciferase-polyA coding sequence replaced exon 1 downstream of the human *TNF* promoter (**b**).

At the DNA level, the coding region of human *TNF* is located within *TNF/LT* locus, together with the lymphotoxin-α (*LTA*), and lymphotoxin-β (*LTB*) genes. Several transcription factor binding sites have been identified in the human *TNF* promoter, in upstream and downstream sequences (Fig. 1a), and all likely contribute to accurate gene regulation through chromatin rearrangement. Successful recombineering of a human *TNF* BAC clone, resulting in the replacement of exon 1 by the luciferase-polyA coding sequence, produced the hTNF.LucBAC construct (Fig. 1b) and was confirmed by PCR screening and pulsed-field gel electrophoresis.

### Expression and activation of luciferase in cell-lines

**Figure 2:**
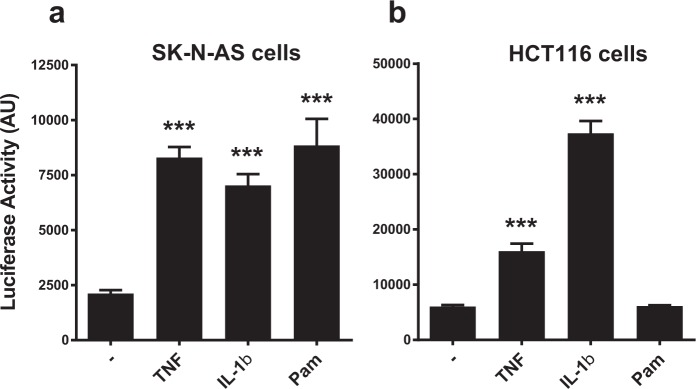
Transient transfection of human cell lines with the hTNF.LucBAC construct. Luciferase activity in SK-N-AS neuroblastoma cells (**a**) and in HCT116 colon carcinoma cells (**b**), unstimulated or stimulated with 100 ng/mL TNF, 20 ng/mL IL-1β or 20 ng/ml Pam_3_CSK_4_ (Pam). Data are representative of three independent experiments, with triplicate cultures per experiment (N = 3, n = 3) and bars represent standard error of the mean. AU = arbitrary units of luminescence. Significant differences from unstimulated cells, ***p < 0.001; ANOVA.

The reporter activity was initially tested on cell-lines by transiently transfecting the hTNF.LucBAC into human neuroblastoma cell-line SK-N-AS, and human colon carcinoma cell-line HCT116. After 24 h transfection, cell-lines were stimulated with different inflammatory activators known to drive *TNF* gene transcription, including TNF itself, IL-1β and the TLR2/TLR1 agonist Pam_3_CSK_4_ (Fig. 2a,b). TNF treatment elicited a similar significant reporter gene response in both cell-types compared to unstimulated cells (p < 0.001, ANOVA). IL-1β also was able to activate luciferase expression in both cell-types, but more potently in HCT116 cells (both p < 0.001). Interestingly, whilst SK-N-AS cells demonstrated luciferase activation in the presence of PAM3CSK4 treatment (p < 0.001), HCT116 cells did not. These data indicate that the hTNF.LucBAC is able to report on *TNF* gene activation *in vitro*.

### Luciferase activity can be induced *in vitro* in a variety of primary cells from the hTNF.LucBAC mouse

Following positive results from transient transfection of the hTNF.LucBAC construct into the neuroblastoma and colon carcinoma cell-lines, we sought to establish a robust, stably integrated system. Linearised hTNF.LucBAC DNA was pronuclear-injected into C57/BL6J mouse embryos and established a mouse strain that expressed luciferase under the control of the human *TNF* promoter. These generated mice had no observable phenotype, nor any sign of developmental abnormalities up to 15 months of age. The hTNF.LucBAC mice produced offspring litters in Mendelian ratios.

**Figure 3:**
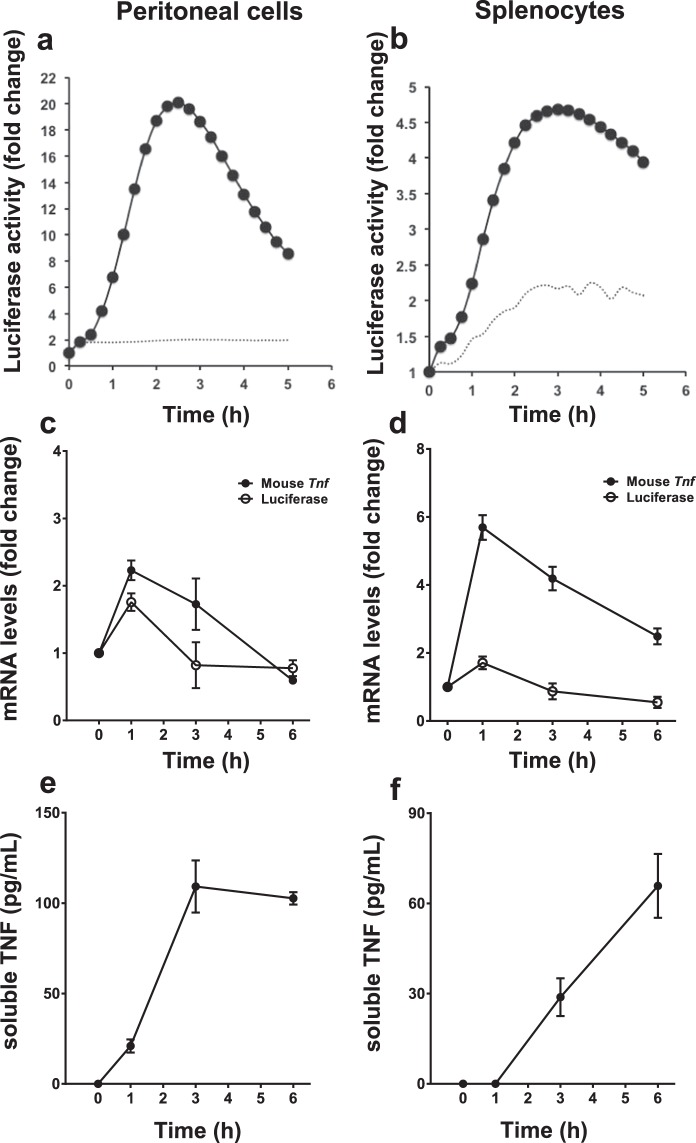
LPS-induced responses of various *ex vivo* primary cell cultures derived from the hTNF.LucBAC mouse strain. Luciferase activity as detected in peritoneal macrophages (**a**) or splenocytes (**b**) without stimulation (dotted line) or after stimulation (solid line) with LPS (10 ng/mL); data are representative of three independent experiments, with triplicate cultures per experiment (N = 3, n = 3). *Tnf* and luciferase mRNA abundance as detected in LPS-stimulated peritoneal macrophages (**c**) and splenocytes (**d**) over 6 h (N = 3 mice). Soluble TNF released to the culture medium in response to LPS treatment of peritoneal macrophages (**e**) and splenocytes (**f**) over 24 h (N = 6 mice). Bars represent standard error of the mean. Significant differences from unstimulated controls, *p < 0.05, **p < 0.01, ***p < 0.001; ANOVA.

Primary cell cultures were established from the hTNF.LucBAC mouse so as to measure the efficiency of the luciferase reporter *ex vivo*. Freshly isolated peritoneal macrophages or splenocytes were cultured and stimulated with 10 ng/mL LPS. Unstimulated cells were used as negative control. Resting peritoneal macrophages showed undetectable luciferase activity, but upon LPS treatment had strong, rapid, dose-dependent activation of the luciferase reporter activity, with an observed peak between 2–3 h (Fig. 3a). Addition of LPS also induced the luciferase reporter activity in splenocyte cultures, albeit to a lower degree, about 5-fold. As with peritoneal macrophages, peak luminescence was observed between 2–3 h (Fig. 3b). To compare the activation of the murine and human *TNF* promoters, mouse *Tnf* and luciferase mRNA levels were assessed by qPCR over a period of 6 h post LPS stimulation. In peritoneal macrophages, peak fold-change in *Tnf* mRNA (2.23 ± 0.15, mean fold change ± s.e.m.), as well as luciferase mRNA (1.76 ± 0.13) occurred at 1 h (Fig. 3c). Similarly, in splenocytes, peak fold-change in *Tnf* mRNA (5.69 ± 0.36), as well as luciferase mRNA (1.71 ± 0.18) also occurred at 1 h (Fig. 3d). Measurement of murine soluble TNF released to the medium over 6 h showed that in peritoneal macrophages and in the splenocytes, the secreted TNF levels reach a plateau phase by 3 h (109.5 pg/ml, and 85.3 pg/ml respectively, Fig. 3e,f). Taken together, the dynamics of mRNA transcription and protein levels of both mouse TNF and luciferase are matched within peritoneal and splenocyte primary cultures.

**Figure 4:**
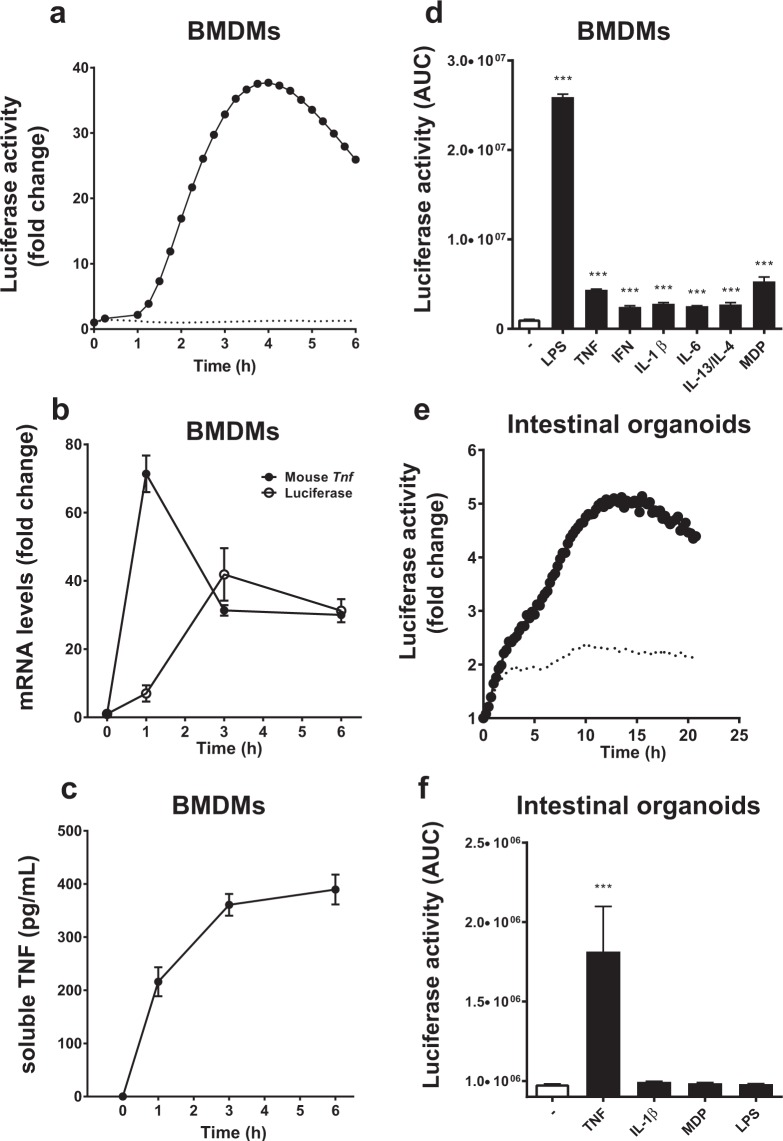
Responses of *in vitro* differentiated cell cultures derived from the hTNF.LucBAC mouse strain. Luciferase activity as detected in BMDMs without stimulation (dotted line) or after stimulation (solid line) with 10 ng/mL LPS; data are representative of three independent experiments, with triplicate cultures per experiment (N = 3, n = 3) (**a**). *Tnf* and luciferase mRNA abundance as detected in LPS-stimulated BMDMs over 6 h (N = 3 mice) (**b**). Soluble TNF released to the culture medium in response to LPS treatment of BMDMs over 24 h (N = 6 mice) (**c**). BMDMs were treated with various ligands and luciferase activity was measured during the cultures (N = 3 mice) (**d**). Luciferase activity as detected in intestinal crypt stem cell-derived organoids without stimulation (dotted line) or after stimulation (solid line) with 100 ng/mL TNF over 24 h (**e**). Organoids were treated with various ligands and luciferase activity was measured during the cultures (N = 3 mice) (**f**); data are representative of three independent experiments, with triplicate cultures per experiment (N = 3, n = 3). Bars represent standard error of the mean. Significant differences from unstimulated controls, **p < 0.01, ***p < 0.001; ANOVA. AUC = area under the curve.

As many immunological methods rely on primary cells that are kept in culture for more than few days, perhaps even for weeks, we tested how longer-term primary cultures behaved with respect to luciferase reporter activation. Here, we used bone marrow-derived macrophages (BMDMs) differentiated *in vitro* for a period of 7–10d, and small intestinal crypt-derived organoids that could survive *in vitro* for several weeks. BMDMs challenged with 10 ng/mL LPS, showed a marked response (40-fold increase) that peaked about 3 h post-stimulation (Fig. 4a). Transcriptional analysis showed that in BMDMs, peak fold-change in *Tnf* mRNA was markedly elevated, occurring at 1 h post LPS stimulation (71.4 ± 5.37), however it was noted that peak fold-change in luciferase mRNA (41.88 ± 7.7) was delayed, occurring at 3 h (Fig. 4b). Nevertheless, luciferase protein expression reflected the observed transcription profile of the human TNF promoter. As seen with peritoneal macrophages, soluble TNF levels released to the medium by BMDM cultures also reached a plateau phase by 3 h (360.7 pg/mL, Fig. 4c). BMDMs were further examined following treatment with various stimuli. Using 20 ng/mL of IL-1β, IL-6, IL-13 and IL-4 in combination or IFN-γ, very low, but significant, levels of luciferase activity was induced compared to unstimulated cells (Fig. 4d); all p < 0.001, ANOVA. For intestinal organoids, 100 ng/mL murine TNF was able to significantly activate luciferase expression, with a different activation profile peaking at 14–15 h post stimulation; p = 0.003, ANOVA (Fig. 4e,f). Other stimuli used to treat organoids, such as IL-1β, MDP and LPS were not observed to drive luciferase reporter activity in these differentiated cultures (Fig. 4f). Overall, these results indicate that the *TNF*-luciferase system reports dynamic ligand- and cell-specific activation profiles in living cells.

### hTNF.LucBAC murine macrophages can be used to study the signalling pathways that regulate human *TNF* gene activation

**Figure 5:**
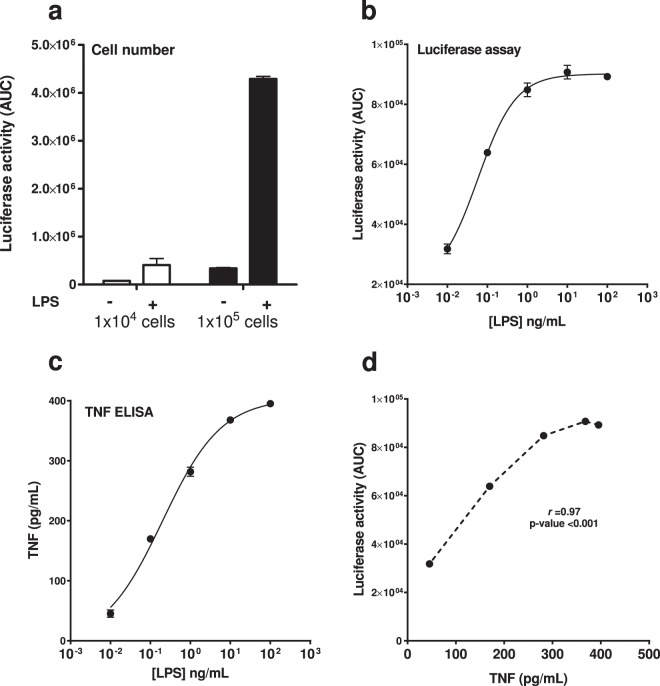
Correlation of LPS-induced luciferase activity with endogenous TNF secretion.  Effect of BMDM cell number on 10 ng/mL LPS-induced luciferase activity; 1 × 10^4^ cells (white bars) and 1 × 10^5^ cells (black bars) **(a)**. Luciferase activity in hTNF.LucBAC BMDMs stimulated with increasing levels of 0.01–100 ng/mL LPS **(b)**. Soluble TNF in the supernatant of treated cells 24 h post-stimulation **(c)**. Pearson’s coefficient of correlation (*r*) was calculated to assess association between TNF with activity of luciferase represented as area under the curve (AUC) (**d**). Data are representative of three independent experiments, with triplicate cultures per experiment (N = 3, n = 3) and bars represent standard error of the mean.

Macrophages produce high amounts of TNF upon their activation. Both in human and in mouse, tumour necrosis factor gene regulation is under tight control and subject to negative feedback mechanisms. BMDMs are an excellent candidate to study such regulatory mechanisms, as they resemble naturally occurring macrophages and they can be produced in high numbers. Moreover, they can be stored as bone marrow stem cells and therefore minimize the number of animals used for research activities. We therefore tested different parameters, such as cell number and LPS dose, to characterise this cell model and its suitability as a reporter system. First, we determined optimal cell number in the live cell luminometry assay. Luciferase activity could be detected in cultures with cell numbers as low as 1 × 10^4^ cells per well, but sensitivity of luciferase detection was increased with a higher cell number (Fig. 5a). Therefore, all subsequent experiments were performed using 1 × 10^5^ cells per well. In BMDMs, a consistent luciferase response was seen following treatment with doses of LPS up to 1 µg/mL. Luciferase activity reached a plateau phase at 10 ng/mL LPS, with higher amounts unable to generate further increase in luciferase activity (Fig. 5b). The EC50 of LPS-induced luciferase activation was calculated to be 0.1 ng/mL. We also observed that the levels of soluble TNF released into the medium 24 h post-stimulation was dependent on LPS dose (Fig. 5c), with a strong positive correlation noted between the BAC-regulated luciferase activity and the endogenous soluble TNF secreted into the culture medium (Fig. 5d). The Pearson’s coefficient of correlation (*r*) of luciferase production to the soluble TNF secreted to culture medium was 0.97. To rule out possible autocrine effects elicited by secreted TNF in LPS-stimulated hTNF.LucBAC BMDMs, we measured luciferase activity in the absence or presence of neutralizing anti-TNFR1/2antibodies. The results showed that there was little, if no effect of blocking the TNFR1 and TNFR2 pathways (Supplementary Information, Table S1).

In our system, LPS clearly induced a very potent luciferase response at 10 ng/mL and therefore we decided to study this pathway in more detail. LPS functions by binding to, and activating, the transmembrane Toll-like receptor 4 (TLR4), which in turn leads to activation of NF-κB and MEK/ERK signalling pathways^18,19^. Both pathways are known to be involved in regulation of the *TNF* gene^20–22^. We tested various inhibitors known to interfere with these signalling pathways. Both the IKK-2 inhibitor BI605906 (10 μM) and the proteasome inhibitor MG132 (10 μM), blocked p65 DNA-binding activity in LPS-stimulated hTNF.LucBAC BMDMs; both p < 0.01 ANOVA (Supplementary Information, Fig. S1a). Similarly, using the MEK inhibitor PD0325901, at 100 nM, we showed complete inhibition of ERK phosphorylation in the same cells stimulated with 10 ng/ml LPS for 15 min; p < 0.001 (Supplementary Information, Fig. S1b).

**Figure 6:**
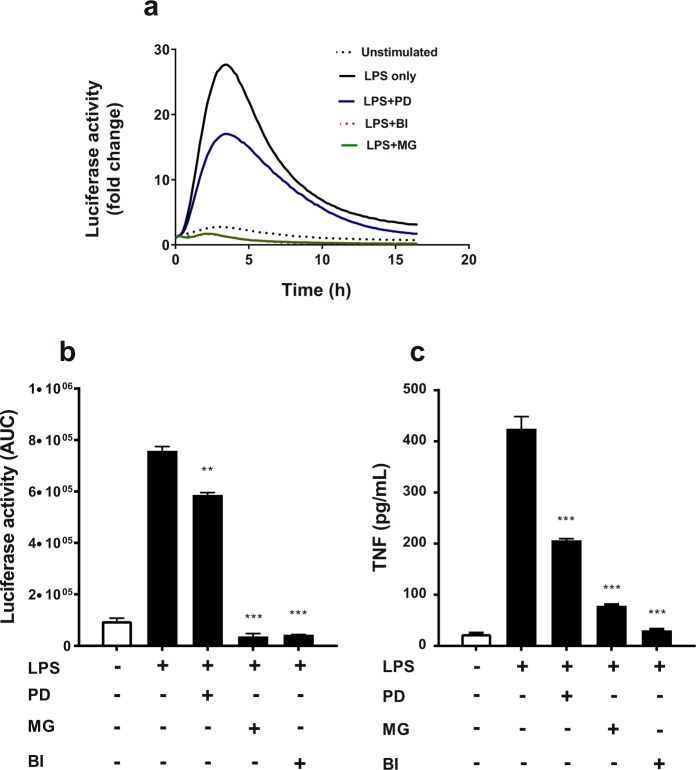
Effect of signal pathway inhibitors on LPS-induced luciferase activity in hTNF.LucBAC BMDMs. Cells left untreated (dotted line) or were stimulated with 10 ng/mL LPS in absence (black line) or presence of MEK inhibitor PD0325901 (PD; blue), proteasome inhibitor MG132 (MG; green) and IKK-2 inhibitor BI605906 (BI; dotted red) (**a**). Luciferase activity represented as area under the curve (AUC) in the absence or presence of each inhibitor in LPS-induced cells (**b**). Soluble TNF in the supernatants of the same cultures, 24 h post-stimulated or untreated BMDMs, in the absence or presence of each inhibitor. TNF levels were undetectable in the presence of each inhibitor alone (**c**). Data are representative of three independent experiments, with triplicate cultures per experiment (N = 3, n = 3) and bars represent standard error of the mean. Significant differences compared to LPS stimulated cells, **p < 0.01, ***p < 0.001; ANOVA.

To test the effect of inhibition of these signalling pathways on the human *TNF* promoter-regulated luciferase activity, hTNF.LucBAC BMDM cultures were pre-incubated in the presence of these inhibitors for 30 min (concentrations as described above), followed by stimulation with 10 ng/mL LPS. Luciferase activity was monitored continuously over a period of 16 h. We observed that MEK inhibition by PD0325901 resulted in ~30% reduction in transgene activation, whereas NF-κB signalling inhibition by BI605906 or MG132 treatment resulted in almost complete inhibition of luciferase activity, all p < 0.01 ANOVA (Fig. 6a,b). At the end of the experiment, soluble TNF was measured by ELISA and we observed similar changes as seen for luciferase activity, with levels of secreted TNF being reduced 50% or more by these inhibitors, p < 0.001 ANOVA (Fig. 6c). This indicates that the regulation of the human *TNF* promoter-luciferase transgene mimics the regulation of the endogenous murine *Tnf* promoter in BMDMs, at least up to 16 h following LPS stimulation.

**Figure 7:**
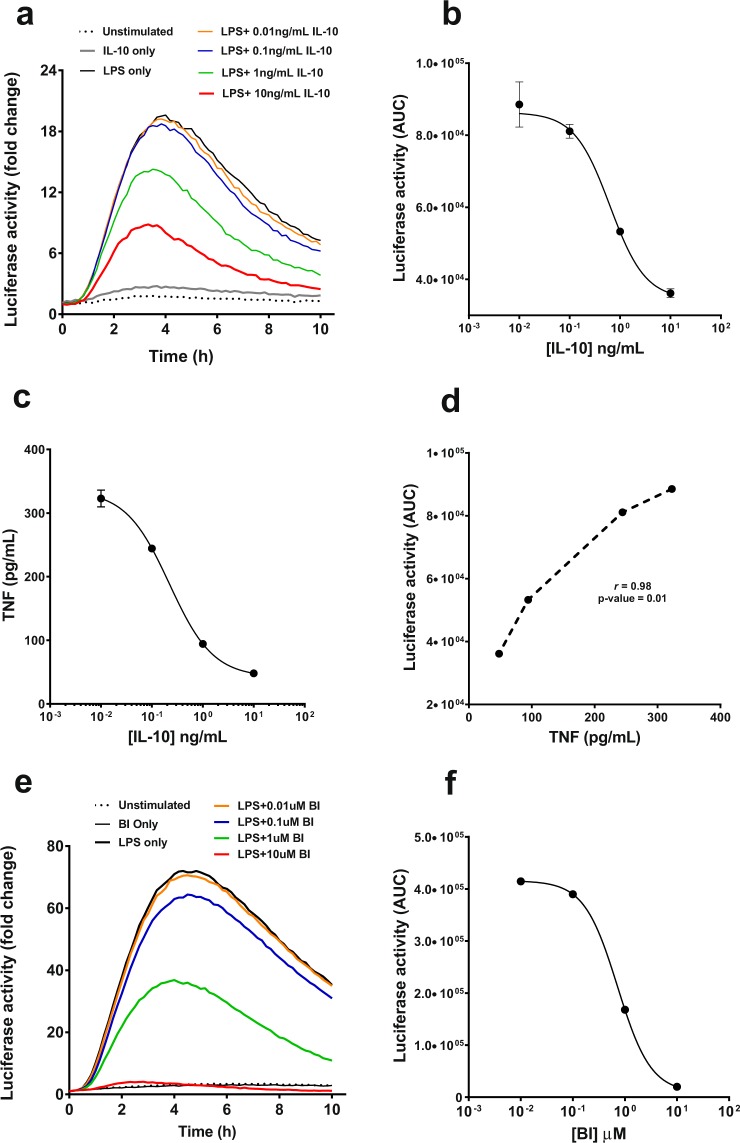
Effect of anti-inflammatory IL-10 on luciferase activity in hTNF.LucBAC BMDMs. BMDMs were either unstimulated (dotted line) or stimulated with 10 ng/mL LPS, in the absence (black line) or in the presence of increasing levels of recombinant murine IL-10, and luciferase activity was monitored over time; IL-10 concentrations 0.01 (orange), 0.1 (blue), 1 (green) and 10 ng/mL (red) (**a**). The ED50 of IL-10 as determined by the dose-dependent inhibition of luciferase activity in stimulated hTNF.LucBAC BMDMs was 0.6 ng/mL (**b**). Soluble TNF detected in the medium 24 h after LPS stimulation in the absence or presence of IL-10 (**c**). Correlation of IL-10 blockade of LPS-induced luciferase activity represented as area under the curve (AUC) with secreted mouse TNF (**d**). IKK-2 inhibitor BI605906 (BI) was used as a positive control for inhibition of luciferase activity and soluble TNF release. BMDMs left unstimulated (dotted line) or treated with 10 ng/mL of LPS, in the absence (black line) or presence of BI inhibitor at 0.01 μM (orange), 0.1 μM (blue), 1 μM (green) and 10 μM (red) (**e**). Luciferase activity (AUC) in the absence or presence of BI in LPS-induced cells **(f**). Data are representative of three independent experiments, with triplicate cultures per experiment (N = 3, n = 3) and bars represent standard error of the mean.

Reporter transgenes are also a useful tool for screening inhibitors of gene regulation. Hence, we tested the luciferase response in hTNF.LucBAC BMDMs to LPS in the presence of different doses of the anti-inflammatory cytokine interleukin 10 (IL-10), a well-established, potent negative regulator of TNF in both monocytes and macrophages^23,24^. Recombinant murine IL-10 (0.1–10 ng/mL) was able to suppress luciferase activation in a dose-dependent manner in 10 ng/mL LPS-stimulated hTNF.LucBAC BMDMs, with IL-10 alone having no effect on luciferase activity; for all doses p < 0.05 ANOVA (Fig. 7a). The calculated ED50 for IL-10 was 0.6 ng/mL (Fig. 7b). The levels of soluble TNF were also reduced by ~50% in the presence of 10 ng/mL IL-10, p < 0.001 (Fig. 7c). The inhibition of luminescence and TNF secretion strongly correlated, with Pearson’s coefficient of correlation (*r*) = 0.98 (Fig. 7d). The IKK-2 inhibitor BI605906, used as a positive control, also showed a clear dose-dependent repression of LPS-induced TNF-luciferase activation, with a calculated IC50 of 0.7 μM (Fig. 7e,f).

## Discussion

In this study, we have generated a novel hTNF.LucBAC construct and a reporter mouse that expresses luciferase under the control of the human *TNF* promoter, and we have examined the response of this transgene in both transfected human cell-lines and primary cells derived from the hTNF.LucBAC transgenic mouse. BACs are large DNA constructs that typically transiently transfect at low efficiencies; however, in two cell-lines tested, SK-N-AS and HCT116, we were able to detect inducible luminescence following stimulation with various ligands that activate the *TNF* promoter, including TNF (signalling through the TNFR1/R2 receptors)^25^ and IL-1β (binding IL-1R)^26^. The TLR1/2 agonist PAM_3_CSK_4_ was also able to induce luciferase expression in SK-N-AS cells, but we observed no reporter gene activation with PAM_3_CSK_4_ in the HCT116 cell-line. HCT116 cells have been reported to lack expression of the TLR1/2 receptor complex^27^, which likely explains the absence of reporter activation. These data clearly indicate that the hTNF.LucBAC construct is functional and can report differential *TNF* gene activation patterns in cultured human cell-lines. Low transfection efficiencies known to occur using large DNA constructs may limit *in vitro* application to other cell-types, including transfection of primary cells, although other suitable vectors may be applied. Importantly, the BAC approach gave us opportunity to stably integrate the construct into the mouse genome, allowing for dynamic assessment of *TNF* gene activation in a more physiological context, and for study of a variety of different cell-types via generation of reporter primary cultures.

To confirm inducible reporter gene expression in our hTNF.LucBAC mouse model, we successfully derived and screened various tissue cell populations and their response to key pro-inflammatory stimuli. Peritoneal macrophages and BMDMs both demonstrated a strong response to LPS, resulting in rapid induction of luminescence with similar maxima of luciferase activity. BMDMs though, showed a delayed relative luciferase peak activity (peak observed at 3–4 h) compared to peritoneal macrophages (peak observed 2–3 h). This delay in BMDMs was also observed at the transcriptional level of the luciferase gene (but not the mouse *Tnf* gene), showing that the luciferase protein expression follows the activation of the hTNF promoter. It has also been reported previously, that peritoneal macrophages and BMDMs have subtly different expression patterns of common cytokine and chemokine receptors, with different gene expression kinetics upon activation with LPS^28,29^. Whilst LPS was consistently the strongest effector, we also showed that the hTNF.LucBAC BMDMs could respond to other pro-inflammatory cytokines (including TNF itself) and to other bacterial effectors, such as MDP. Furthermore, using hTNF.LucBAC isolated splenocytes, a mixed population consisting mainly of T- and B-lymphocytes as well as macrophages, we also observed the same rapid induction of luciferase with lower overall fold induction. The latter observation was likely because only B-lymphocytes, which represent 40–50% of total splenocytes, can respond to LPS in such an experimental setting. However, the doses of LPS we used in this study were far lower than those previous reported for *in vitro* B-lymphocyte activation^30–32^ and as a result lack of luciferase induction is to be expected. It is also worth noting that unstimulated splenocyte cultures showed elevated luciferase activity. This is probably because the various cell-types within this mixed population, cannot reach a resting status as they likely interact with each other, elevating basal levels of stimulatory molecules such as IL-12, TNF and IL-6, each of which can enhance *TNF* promoter activity^28,33^.

A key new technology in biological research has been the development of 3D organoid cultures^34^. Intestinal organoids have been proposed to be an excellent *ex vivo* model for study of inflammatory bowel disease (IBD)^35^. IBD is a chronic inflammatory condition of the gastrointestinal tract where TNF is a major proinflammatory molecule involved in both development and progression of the disease, and therefore represents an important biological target for drug development^36^. We therefore derived mouse intestinal crypt stem cells and differentiated them to 3D intestinal epithelium organoid cultures in order to test our reporter system. We observed induction of luciferase activity in hTNF.LucBAC organoid cultures in response to TNF, peaking at about 14 h post-stimulation. Unlike BMDMs, other stimuli such as IL-1β and bacterial products were unable to elicit a reporter response in intestinal organoids. Overall, these results highlight the utility of this transgenic mouse model, where cell-types of interest can be isolated to specifically test stimulation response patterns to physiological stimuli and to pathological changes.

In BMDMs, the most potent effector ligand was LPS, followed by TNF and MDP, respectively. It is well established that LPS is a major inducer of inflammatory response in immune cells, leading to rapid expression and secretion of pro-inflammatory cytokines^37^, whilst in non-immune cells (such as intestinal epithelial cells), the *TNF* promoter is activated by TNF alone, in an autocrine way, as previously reported^38^. BMDMs are an ideal model for inflammation research as they can be obtained in high yield, with homogeneity and a long lifespan in culture^28^, and are strongly responsive to a variety of physiological stimuli. We measured both dynamic luciferase activity in LPS-stimulated hTNF.LucBAC BMDMs and their secretion of soluble murine TNF and found a strong positive linear correlation, suggesting that the regulation of the human *TNF*-luciferase transgene was indeed similar to regulation of the endogenous murine *Tnf* promoter. Our hTNF.LucBAC luciferase reporter system therefore allows for measurement of real-time gene activation in living cells as a response to pro-inflammatory stimuli. The reporter data described here, together with our transcription-based and secretion-based end-point assays, supports previous work using transcriptomic approaches^25,39^.

Key regulators of the tumour necrosis factor promoter are members of the NF-κB family, transcription factors that share a highly conserved DNA-binding domain known as the Rel homology domain (RHD)^40^. Activation of transmembrane receptors by external stimuli ultimately signal to the IKK complex. IKK-2 subsequently phosphorylate IκBs, a family of inhibitor proteins that retain NF-κB dimers within the cytoplasm in an inactive state. Phosphorylated IκBs are then targeted for ubiquitination and degradation by the proteasome which releases, NF-κB dimers to translocate into the nucleus and activate their target genes. Inhibitors of NF-κB have been developed targeting this key signalling pathway, and NF-κB has long been thought as a potential therapeutic drug target^41–43^. We tested whether BMDMs derived from our mouse model could be used to assay known inhibitors of this pathway. Using potent inhibitors of IKK-2 (BI605906) and the proteasome (MG132), we were able to show significant blockade of the induced luciferase activity and inhibition of p65 DNA-binding activity in LPS-stimulated BMDMs obtained from the hTNF.LucBAC reporter mouse. Our inhibition data is in agreement with that of a previous study which showed similar levels of inhibition of NF-κB DNA-binding by MG132 in rat renal tubular epithelial cell-line stably transfected with the pNF-κB-Luc reporter construct^44^. Here we also tested the inhibitor PD0325901 which is known to block MEK phosphorylation. Treatment of hTNF.LucBAC BMDMs with PD0325901 showed complete blockade of ERK phosphorylation, decreased LPS-induced luciferase activity and reduced secretion of soluble mouse TNF. Previous studies using this inhibitor and genetic approaches (*Map3k8*^−/−^ and Map3k8 kinase-dead mice) have revealed that the TPL2/MEK/ERK pathway is involved in the regulation of TNF production in macrophages^20^.

One of the most potent natural inhibitors of TNF is Interleukin-10 (IL-10). IL-10 is an immunoregulatory cytokine that plays an essential role in suppressing pro-inflammatory molecules such as TNF and other cytokines, including IL-6 and IL-1β. In our study, we observed that IL-10 could indeed supress the transgene-regulated luciferase activation of LPS-stimulated hTNF.LucBAC BMDMs and reduce release of soluble TNF to the culture medium. In support, Denys and colleagues have previously reported that IL-10 could supress TNF production in primary human macrophages^45^, and a later study by Smallie *et al*. also showed that IL-10 could inhibit transcriptional elongation of *TNF* in LPS-treated human macrophages by blocking RelA recruitment to a sequence downstream of the *TNF* 3′ untranslated region^46^. Previous use of an adenovirus-based system to express *TNF* promoter-luciferase reporters of varying, but limited, sizes (~1000 bp) had shown LPS-induced expression can only be mildly attenuated by IL-10^46^. Whilst exogenous expression systems such as these are flexible, and do not require the maintenance of a transgenic mouse colony, they suffer from a lack of regulatory context. Our hTNF.LucBAC mouse model however, shows far greater repression of LPS-induced luciferase activity by IL-10 over an extended time period using live cell luminometry. This likely reflects the more physiological situation in which the regulation and function of the human *TNF* promoter is studied.

The use of a BAC as the basis of our reporter system is vital as it ensures all known and as-yet unknown regulatory elements involved in *TNF* gene activation are maintained. In this study, the construct responded to a variety of known pro- and anti-inflammatory stimulations. This model could easily be applied in screening for pro- and anti-inflammatory molecules in tissue specific *ex vivo* organotypic culture systems, such as intestinal organoids as demonstrated here. With access to high-sensitivity bioluminescence imaging systems, it is plausible that this model could be used for live *in vivo* analysis of inflammatory modulators.

Carlsen *et al*. previously generated a NF-κB reporter mouse^47^. This mouse model has a randomly integrated DNA construct consisting of three NF-κB binding sites from the Ig light chain promoter driving luciferase expression, which served as good reporter of NF-κB activation *ex vivo* and *in vivo*. By imaging anaesthetised NF-κB reporter mice, Carlsen and colleagues were able to visualise luminescent reporter signal after immune challenge^47^. Our interest lies in the regulation of the human *TNF* gene, which is affected by several signalling pathways and transcription factors. Therefore, the human *TNF* promoter requires expression from a recombinant BAC to fully contain all critical regulatory sites and in correct positional context. In future experiments it would be interesting to study the dynamics and reporter intensity of our BAC-based system. In conclusion, we have generated a valuable reporter system for studying the regulation of the human *TNF* promoter in different cell-types and tissues. Future experiments will reveal whether the hTNF.LucBAC mouse strain can be used to study the regulation of TNF synthesis in the context of both health and disease, and to assess potential therapeutic targets/interventions.

## Materials and Methods

### Generation of hTNF.LucBAC construct

The human *TNF* BAC clone (RP11-184F16; obtained from Life Technologies, UK) was purified using a Machery-Nagel Nucleobond BAC100 kit and used to transform electro-competent SW102 *E. coli* (a kind gift from Neal Copeland; Houston Methodist Research Institute, Houston, TX, USA). SW102-*TNF* positive clones were confirmed by pulsed-field gel electrophoresis analysis. Recombineering was performed using seamLess *GalK* mediated selection/counter selection strategy, as described previously^26,48^. In brief, two targeting cassettes designed to excise the first coding exon of the *TNF* gene were generated to first incorporate the *GalK* gene within the BAC, positive recombinants derived by selection on minimal media containing galactose, followed by the removal of *GalK* and integration of the luciferase-polyA coding sequence from pGL3Basic (Promega, UK) and counter selection using 2-deoxy-galactose and glycerol. Correctly modified colonies were identified by PCR screening and pulsed-field gel electrophoresis.

### Generation of hTNF.LucBAC transgenic mouse

The hTNF.LucBAC construct was purified, linearized by BsiWI restriction enzyme digestion and subjected to Sepharose 4B-CL column purification. Linear DNA was then microinjected into the pronucleus of one-day single cell mouse embryos from B6D2F1 mice (Envigo). Zygotes were cultured overnight, and the resulting 2 cell embryos surgically implanted into the oviduct of day 0.5 post-coitum pseudo-pregnant mice. After birth and weaning, genomic DNA was extracted using RED Extract-N-AmpTM Tissue PCR Kit (XNAT; Sigma-Aldrich, UK) and used to genotype pups. Founders with luciferase gene integration were back crossed onto C57Bl/6JOlaHsd and transgene positive mice derived from the founders were used in all subsequent experiments. All experimental procedures and protocols were carried out in accordance to the guidelines of the Animals (Scientific Procedures) Act 1986, and subject to local ethical approval by the University of Manchester Animal Welfare and Ethical Review Board (Project licence number 70/7800).

### Genotyping

Genomic DNA extracted using RED Extract-N-AmpTM Tissue PCR Kit (XNAT, Sigma) and stored at −20 °C was used for genotyping by Polymerase Chain Reaction (PCR). The PCR product generated was a result of amplification of a region of the human *TNF* promoter gene by using 250 nmol/L PCR primers as follows: h*TNF*-F: GGGCAGCTCCAGTGGCTGA and h*TNF*-R: GGTAGGAGACGGCGATGCGG. The PCR conditions were as set up initial denaturation step at 95 °C for 30 s, followed by 35 cycles of denaturation at 95 °C for 30 s, annealing at 60 °C for 30 s, and extension at 68 °C for 60 s. We used a PTC-225 Peltier Thermal Cycler (MJ Research) for all PCR reactions.

### Transient transfection of hTNF.LucBAC in human cell lines

The human neuroblastoma cell-line SK-N-AS (from ECACC, 94092302) and human colon carcinoma cell-line HCT116 (from the ATCC^®^; CCL-247^™^) were cultured in MEM and DMEM respectively, supplemented with 10% v/v foetal calf serum (Gibco, UK) and 1% w/v non-essential amino acids (Sigma-Aldrich). Both cell-lines were sub-cultured at densities between 80–90%. Cells were routinely tested by PCR to exclude mycoplasma contamination. For transient transfection, BAC DNA was purified (BAC100 Nucleobond kit, Macherey-Nagel, Germany) and 3 μg was used to transfect 10^6^ cells in a 10 cm dish using 9.87 µL ExGen500 transfection reagent (following manufacturer’s recommendations). For luminometry, cells were split and 5 × 10^5^ cells were re-seeded in 24 well luminometry plates 24 h prior to stimulation.

### *In vitro* generation and culture of macrophages

Mice were culled by cervical dislocation and resident peritoneal macrophages were harvested following intraperitoneal injection of mice with 4 mL of RPMI medium supplemented with 10% v/v foetal bovine serum (FBS) and subsequent aspiration. Macrophages were purified from the resulting cell suspension by adherence after 3 h of culture at 37 °C and removal of non-adherent cells by washing with phosphate-buffered saline (PBS). Bone marrow-derived macrophages (BMDM) were isolated and prepared as described previously^49^. Briefly, bone marrow cells were plated in 10 mL of complete BMDM medium (RPMI 1640 medium [Sigma] supplemented with 10% v/v FBS, antibiotics, 50 ng/mL mouse colony stimulating factor (MCSF), and 50 μM β-mercaptoethanol) at 5 × 10^6^ cells per 90 mm bacterial petri dish (Sterilin, UK). After 4d of culture, 10 mL of complete BMDM medium was added, and cells were cultured a further 3d. Non-adherent cells were aspirated, and remaining adherent cells were harvested by incubation with 5 mL of PBS supplemented with 5% v/v FBS and 2.5 mM EDTA. The purity of BMDMs were >95% F4/80 positive as determined by flow cytometry. For protein chemistry experiments, BMDMs were cultured as 1 × 10^6^ cells/well, in 6-well plates in 2 mL medium and they were left to rest overnight. The next day, the cells were stimulated with 10 ng/mL LPS for the indicated time points and at the end of stimulation, they were washed once in cold PBS and lysed in 250 μL RIPA buffer (Sigma) for 15 min on ice. After centrifugation at 10000 × *g* for 10 min, the clear supernatant was transferred into new Eppendorf tubes and stored at −80 °C until further use. For detection of ERK phosphorylation and total ERK protein, the ERK1/2 (pT202/Y204 + Total) ELISA Kit (ab176660; AbCam, UK) was used. For detection of p65 DNA-binding activity, the TransAm Flexi NFκB p50/NFκB p65 ELISA kit (Active motif; UK) was used.

### *G*eneration and culture of splenocytes *in vitro*

Single-cell suspensions were generated from spleen via gentle homogenization through nylon mesh filters (70 µM; Becton Dickinson, UK). Erythrocytes in spleens were lysed with ammonium chloride potassium (ACK) lysis buffer and the cell pellet was resuspended in DMEM medium supplemented with 10% v/v/FBS, 1% w/v/Penicillin/Streptomycin, 1 mM glutamine and 50 μM β-mercaptoethanol.

### Small intestinal organoid culture

Small intestinal crypts were isolated as described previously^50^. Briefly, proximal small intestine tissue was harvested and flushed with ice-cold sterile PBS to remove luminal contents. Intestine was then opened longitudinally and cut into 1–2 cm pieces followed by washing in cold PBS until the supernatant was clear. Tissue fragments were then incubated with 2 mM EDTA for 30 min with gentle shaking. After removal of buffer containing EDTA, the fragments were resuspended in shaking buffer (43.3 mM sucrose and 59.4 mM sorbitol in PBS) and vigorously shaken for 2 min to ensure crypt release. Crypt-containing supernatant fractions were pooled together, passed through a 70 μm cell strainer, and centrifuged at 200 × *g* for 10 min at 4 °C. The crypt pellet was resuspended in ice-cold Matrigel (Scientific Laboratory Supplies, UK) (500 crypts/50 µL Matrigel) containing 50 mg/mL recombinant mouse epidermal growth factor (EGF), 100 ng/mL recombinant mouse Noggin CF and 500 ng/mL recombinant mouse R-spondin 1; all obtained from R&D Systems UK. Using pre-chilled pipette tips, the crypt/matrigel suspension was plated as 50 µL/well in a 24-well plate (Nunc) and incubated for 5–10 min at 37 °C for the Matrigel to polymerize. Subsequently, 0.5 mL culture medium was added per well (Advanced DMEM/F12 containing 1% w/v L-glutamine, 1% w/v penicillin/streptomycin, 10 mM HEPES and 1% v/v N2 supplement and 0.5% v/v B27 serum-free supplement (Thermo Scientific, UK). Small intestinal organoids were cultured for 7 d and were split once before they were used for experiments at day 10.

### Real-time PCR

Freshly isolated peritoneal macrophages and splenocytes, and terminally differentiated BMDMs, were used for transcriptional studies. Cells were either left unstimulated or stimulated with LPS (at 10 ng/mL for macrophages and 1 µg/mL for splenocytes) at various time points up to 6 h. Total RNA was purified using the RNeasy mini kit (Qiagen, UK) and isolated RNA converted to cDNA using the High Capacity RNA-to-cDNA Kit (Applied Biosystems, UK). For real-time PCR reactions, the Taqman Fast Advanced Master mix was used, together with FAM probes for mouse *Tnf* (Mm00443258_m1), luciferase (Mr03987587_mr) and hypoxanthine guanine phosphoribosyltransferase (*Hprt*; Mm00446968_m1). The reactions were run using a LightCycler® 480 qPCR Instrument (Roche, UK) and the data were analysed according to the ΔΔCt method^51^.

### Luciferase reporter assay

For luciferase assay cells were cultured in 96 well plates (OptiPlate-96, White Opaque 96-well Microplate; Perkin Elmer, UK) in 0.2 mL medium containing 1 mM luciferin (Promega). Cells were stimulated with 10 ng/mL LPS (*Salmonella enterica* serovar Minnesota R595; Alexis Biochemicals, UK). The luciferase activity was measured over time in a CO_2_ Lumistar Omega luminometer (BMG Labtech, UK). Unstimulated cells were used as negative control. Recombinant murine cytokines also tested included 0.01–10 ng/mL IL-10 (Peprotech, UK), 20 ng/mL IL-1β (Shenandoah Biotechnology, USA), 20 ng/ml Pam_3_CSK_4_ (Sigma), 50–100 ng/mL TNF (PeproTech), 20 ng/mL of IL-4, IL-6, IL-13 and IFN-γ (PeproTech). Bacterial N-acetylmuramyl-L-alanyl-D-isoglutamine hydrate (i.e. muramyl dipeptide, MDP (Sigma)) was tested at 500 ng/mL. NF-κB and ERK signalling pathway inhibitors used included BI605906 (MedChemExpress, USA) and MG132 (Sigma), both used at 10 µM; and PD0325901 (Sigma) used at 100 nM. For TNFR blocking experiments, we used purified anti-mouse CD120a (TNFR1/p55) neutralizing antibody (clone 55R-170, Biolegend UK Ltd), purified anti-mouse CD120b (TNFR2/p75) neutralizing antibody (clone TR75-32.4-170, Biolegend UK Ltd), anti-human TNF RI/TNFRSF1A neutralizing antibody (mab225, R&D Systems) and the Armenian Hamster IgG isotype control antibody, Functional Grade (eBioscience) at final concentration of 50 μg/ml.

### Statistical analysis

Results are expressed as mean ± standard error of mean (SEM). Following assessment for normality and equality of variances, statistical inferences on data were performed using one-way analysis of variance (ANOVA) followed by pairwise comparisons of treatment means using Dunnett’s post-hoc test (vs. unstimulated controls or vs. stimuli used in the inhibition studies). Differences were considered statistically significant when p < 0.05. Luciferase activity represented as area under the curve (AUC) and soluble TNF secreted levels were evaluated using Pearson’s correlation analysis. Statistical analyses were performed using GraphPad Prism 7 Software Statistical Package, La Jolla CA; USA.

## Supplementary information

Supplementary Information

## Data Availability

All data generated or analysed during this study are included in this published article (and its Supplementary Information files).
