# Supplementary material for: Human TNF-Luc reporter mouse: A new model to quantify inflammatory responses
Source: Sci Rep. 2019 Jan 17;9:193. doi: 10.1038/s41598-018-36969-x (PMC6336827; doi:10.1038/s41598-018-36969-x)
Supplement: Supplementary file 1 — Supplementary Information [file 41598_2018_36969_MOESM1_ESM.pdf]

Human TNF-Luc reporter mouse: A new model for measuring inflammatory responses. Faisal Minshawi, Mike White, Werner Muller, Neil Humphreys, Dean Jackson, Barry J. Campbell, Antony Adamson, and Stamatia Papoutsopoulou.

Supplementary Information

Supplementary Table S1: **Blocking of TNFR1 or TNFR2 does not affect the human *TNF* promoter-induced luciferase activity in lipopolysaccharide stimulated hTNF.LucBAC cells.** Differentiated bone marrow-derived macrophages (BMDMs) or isolated splenocytes were pre-treated for 4h with either 50µg/mL isotype control antibody or neutralizing anti-TNFR1 or anti-TNFR2 antibodies prior to stimulation with 10ng/mL LPS (N=3-5 mice, with triplicate samples). Luciferase activity was monitored for 16h and expressed as mean area under the curve (AUC) ± standard error of mean (s.e.m.).

	**BMDMs**	**Splenocytes**
**Unstimulated cells**	41320 ±1656	24604 ± 334
**LPS stimulated cells**	1169169 ± 51884	107095 ± 21735
**LPS + Isotype control antibody**	870843 ± 71848	115323 ± 27866
**LPS + anti-murine TNFR1 antibody**	1027788 ± 55856	103326 ± 21070
**LPS + anti-humanTNFR1 antibody**	1080763 ± 56131	122319 ± 24259
**LPS + anti-murine TNFR2 antibody**	1017231 ± 22554	102298 ± 4825

Human TNF-Luc reporter mouse: A new model for measuring inflammatory responses. Faisal Minshawi, Mike White, Werner Muller, Neil Humphreys, Dean Jackson, Barry J. Campbell, Antony Adamson, and Stamatia Papoutsopoulou.

Supplementary Figure S1: **Effect of NF-κB and MEK/ERK pathway inhibitors on lipopolysaccharide-induced hTNF.LucBAC BMDMs.**

(a) Cells were left untreated or pre-treated for 30min with the IKK-2 inhibitor BI605906 or the proteasome inhibitor MG132 (both at 10µM), followed by stimulation with 10ng/mL LPS for 30min. At the end of the stimulation, cells were lysed with RIPA buffer and the cleared lysates were used for ELISA. p65 DNA binding activity was blocked by both inhibitors (**p<0.01 and ***p<0.001, ANOVA; N=6 mice). **(b)** Cells were pre-treated in the absence or presence of the MEK inhibitor PD0325901 (for 30min, at 100nM) and then stimulated with 10ng/mL LPS for 15min. phospho-ERK/total ERK was measured in lysates by ELISA. Phosphorylation of ERK was completely blocked by PD0325901 (***p<0.001, ANOVA; N=6).

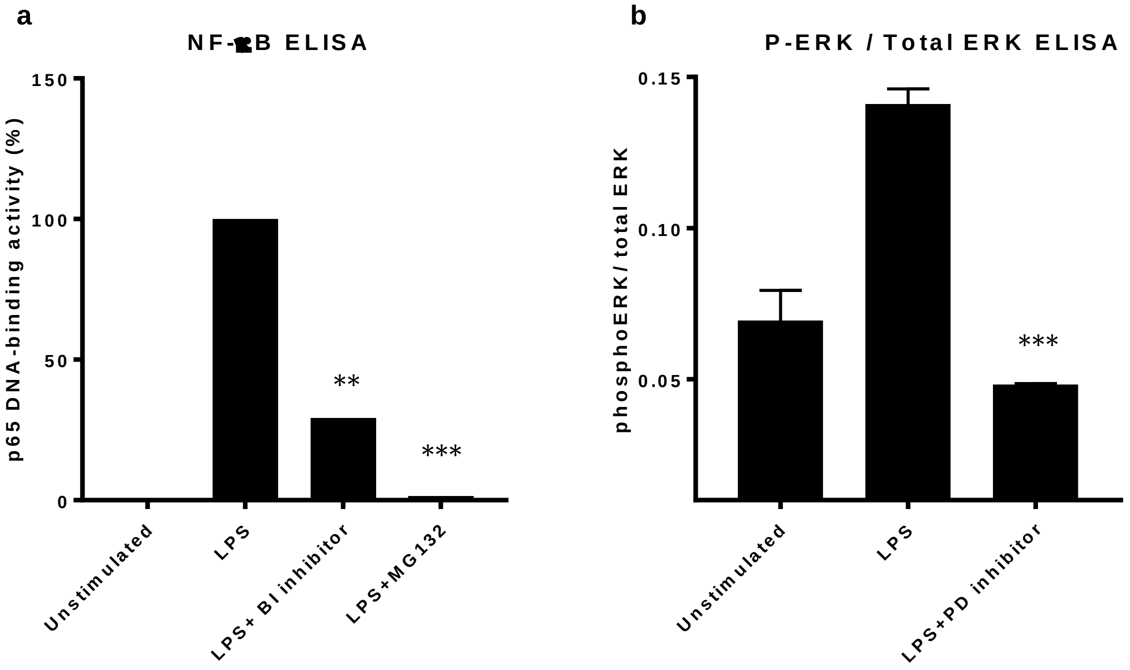

Human TNF-Luc reporter mouse: A new model for measuring inflammatory responses. Faisal Minshawi, Mike White, Werner Muller, Neil Humphreys, Dean Jackson, Barry J. Campbell, Antony Adamson, and Stamatia Papoutsopoulou.
